# Social distancing and severe acute respiratory syndrome coronavirus 2 transmission: A case study from Araraquara, São Paulo, Brazil

**DOI:** 10.1590/0037-8682-0197-2021

**Published:** 2021-06-02

**Authors:** Dalson Britto Figueiredo, Lucas Emanuel de Oliveira Silva

**Affiliations:** 1 Universidade Federal de Pernambuco, Departamento de Ciência Política, Recife, PE, Brasil.; 2 Universidade Estadual de Ciências da Saúde de Alagoas, Maceió, AL, Brasil.

**Keywords:** SARS-CoV-2, COVID-19, Lockdown, Social distance

## Abstract

**INTRODUCTION:**

This study evaluates the impact of social distancing on the spread of coronavirus disease (COVID-19).

**METHODS:**

Using data from the Brazilian Ministry of Health, we conducted an interrupted time series analysis to estimate the impact of lockdown on the number of daily cases of COVID-19 in Araraquara, São Paulo.

**RESULTS:**

Policy changes neutralized the positive trend of the disease. To provide more reliable evidence, we added two control cases from Araçatuba and São Carlos to the regression model, and the results remained consistent.

**CONCLUSIONS:**

Social distancing interventions are effective tools for flattening epidemic curves.

Official data from the Ministry of Health indicate that Brazil has one of the fastest-growing coronavirus disease (COVID-19) epidemics globally, with more than 14 million registered cases and more than 390,000 deaths^1^. Current estimates place Brazil as the second leading contributor to the total death toll, and it is just behind the United States, where the number of fatalities surpassed 564,000^2^.

In the absence of vaccines and antiviral treatment, the most effective strategy for curbing the spread of the virus is the adoption of non-pharmaceutical interventions (NPIs)^3^. In particular, social distancing policies may include the cancelation of small gatherings, the closure of educational institutions, internal mobility control, mass agglomeration prohibition, *cordon sanitaire*, public transport restrictions, and forced quarantine^3^. 

In Brazil, institutional responses to the COVID-19 epidemic have been characterized by a lack of coordination among national, state, and local administration^4^. Additionally, President Jair Bolsonaro has repeatedly minimized the destructive power of the new virus and challenged the role of social distancing interventions^5^ ^,^ ^6^. The current political instability is another major obstacle to mitigating the spread of COVID-19; in less than a year, Brazil has had four different ministries of health. 

In March 2020, the Brazilian Superior Federal Court, in a unanimous decision, ruled that states and municipalities are constitutionally entitled to implement public policies to control the new coronavirus^7^. On February 12, 2021, the Araraquara City Council issued a decree (number 12.485), which defined stricter social distancing measures at the local level^8^. In this study, we used an interrupted time-series model to evaluate the effect of lockdown on the spread of COVID-19. To ensure more robust evidence, we included two case controls from Araçatuba and São Carlos to facilitate the estimation of what would be observed in the absence of the intervention. 

We collected official information from the Brazilian Ministry of Health website, which provides updated epidemiological data on the COVID-19 epidemic^1^. The time series began on April 2, 2020, and ended on March 16, 2021. From the Araraquara City Council website, we gathered information on more restrictive social distancing policies adopted from February 21, 2021, to March 2, 2021. We defined a two-week observation window after the end of the lockdown period. Using an interrupted time-series model, we examined the trend of new cases before and after the policy change.

We complemented the statistical analyses with two control cases: Araçatuba and São Carlos. The reason for including a control was to minimize the potential confounding variables (history bias and concurrent events)^9^. The case-control selection followed two criteria: (a) statistical similarity and (b) location characteristics. Regarding the epidemic curve, Araçatuba and Araraquara had similar trends before the policy change, which suggests that both cities are comparable $\left(\beta_{1_{Araraquara}}=\;.14;\;\beta_{1_{Araçatuba}}=.18\right)$. The inclusion of São Carlos was justified by the location-characteristic-based control^9^. Araraquara and São Carlos are border municipalities with similar population sizes (while Araraquara has 238,339 inhabitants, São Carlos has 254,484).

The equation for the full model, including the controls, was estimated as follows:

$$Y_{jkt}\;=\;\beta_{0}\;+\;\beta_{1}{time\;}_{t}+\beta_{2}{level}_{t}+\;\beta_{3}{trend}_{jt}+\;\beta_{4}G_{k}+\beta_{5}G_{k}{time}_{t}+\beta_{6}G_{k}{level}_{jt}\;+\;\beta_{7}G_{k}{trend}_{jt}\;+\;\varepsilon_{jkt}$$

The intercept, β_0,_ informs the expected value of the dependent variable for the control group before the intervention. β_1_ describes the trend of the series for the control group before the institutional change, while β_2_ captures the change in level for the control group. β_3_ represents the change in the trend in the control group after lockdown. β_4_, the coefficient associated with the dummy variable, G, indicates the difference between the treatment and control groups before the institutional change. β_5_, β_6_, and β_7_ capture the interaction between the variables of time, level, and trend with the group indicator. In substantive terms, β_5_ represents the difference in the series trend before the intervention between the treatment and control groups. β_6_ can be interpreted as the difference in the series level immediately after the intervention. The last coefficient of interest is β_7_, which indicates the difference in the trend of the series during the period after the implementation of the social mobility restriction policy for the treatment group in relation to the control group. 

Replication materials, including raw data and computational scripts, are publicly available at: https://osf.io/jpt4e/. All the statistical analyses were performed using *R Statistical*, version 4.0.5.

Figure 1 shows the moving average of new cases of COVID-19 and transmission rate in Araraquara. The light blue vertical interval indicates the length of the lockdown (number of days). The trend before the intervention was positive (β_1_ = 0.14, p <.001). After the policy change, we observed a shift in the coefficient sign (β_3_ = -3.36, p-value <.001), which means that social distancing effectively neutralized the spread of COVID-19 in Araraquara see regression coefficients in Supplementary Material (Table 1). In addition, the COVID-19 transmission rate decreased by 39.5% during the lockdown, and current estimates suggest an Rt lower than one (see Figure 1B).

**FIGURE 1:**
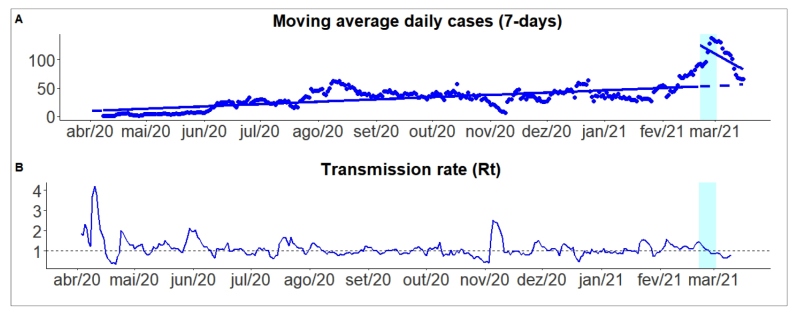
Moving average number of new cases of Covid-19 and transmission rate in Araraquara, São Paulo, Brazil.

Figure 2 compares the trend of new COVID-19 infections in Araraquara (treated case) and Araçatuba (control case). The comparative analysis over time facilitates the visualization of what we should have observed in Araraquara in the absence of the intervention. Both cities had very similar trends before the lockdown see regression coefficients in Supplementary Material (Table 2). Araçatuba did not implement more restrictive social distancing measures, while Araraquara did. The transmission rate in Araraquara decreased sharply, while Araçatuba kept it above one during the two-week observation window after the lockdown (see Figure 2B). 

**FIGURE 2:**
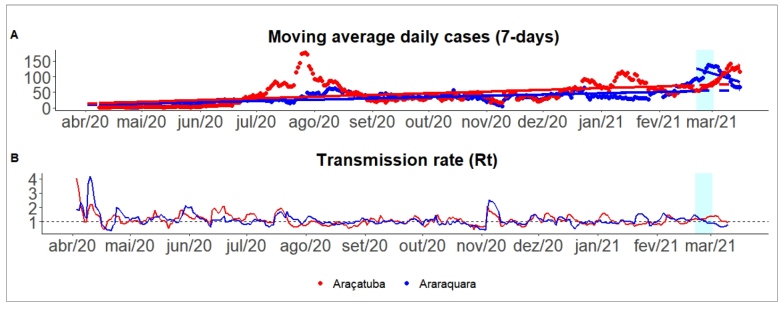
New cases of Covid-19 and transmission rate in Araraquara and Araçatuba, São Paulo, Brazil.

Similar to Araçatuba, São Carlos did not implement a lockdown during the same period. Araraquara treatment resulted in a reduction in the number of cases and transmission. Conversely, São Carlos maintained a transmission rate above one during the two-week observation window (see Figure 3B). See regression coefficients in Supplementary Material (Table 3).

**FIGURE 3:**
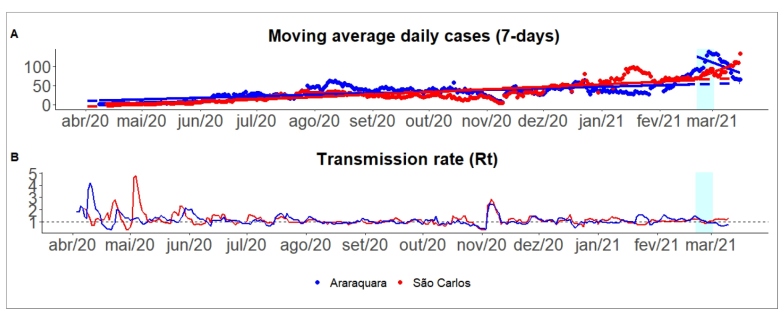
New cases of COVID-19 and transmission rate in Araraquara and São Carlos, São Paulo, Brazil.

Evaluating the impact of NPIs is vital for evidence-based public policy. To our knowledge, this study provides the first systematic confirmation that social distancing policies adopted in Araraquara, São Paulo, were effective in mitigating the spread of COVID-19. We conducted a series of robustness tests and concluded that these results were not driven by specific measures of the outcome variable (daily number of cases or transmission rate). Using data from 152 countries and a stochastic continuous-time Markov chain (CTMC) model, Oraby et al. found that well-timed lockdowns can reduce pandemic peak incidence by splitting hospitalizations over time^10^. Haug et al. examined the impact of 6,068 NPIs implemented in 79 territories and found that the most effective interventions included curfews, lockdowns, and restricting the agglomeration of people^3^. Using data from China, Figueiredo et al. found that social distancing policies were effective in decreasing COVID-19 incidence and mortality rates^11^. Based on a Poisson regression model, Vokó and Pitter examined data from 28 countries and found that national lockdown policies contributed to the suppression of the COVID-19 pandemic in Europe^12^. In Brazil, Silva et al. employed interrupted time series to estimate the impact of lockdowns in four Brazilian state capitals (Belém, Ceará, Recife, and São Luís)^13^. They reported that strict social distance measures reduced the spread of COVID-19. Our findings support these results and highlight the role of social distancing as a critical tool for flattening the epidemic curve. 

## SUPPLEMENTARY MATERIAL

**TABLE 1:** Regression coefficients of the ITS model for Araraquara.

	coefficients Ω
(Intercept)	8.37 * (3.66)
β_1_	0.14 *** (0.02)
β_2_	82.04 *** (14.34)
β_3_	-3.36 *** (0.97)
nobs	349
r.squared	0.31
adj.r.squared	0.30
sigma	32.91
statistic	51.29
p.value	0.00
df	4.00
*** p < 0.001; ** p < 0.01; * p < 0.05.	

Ω β_1_ describes the existing trend in COVID-19 daily cases, β_2_ informs the level change immediately after the policy change, and β_3_ indicates the slope change after the lockdown. Theoretically, we expected that β_3_ is less than 0.

## SUPPLEMENTARY MATERIAL

**TABLE 2:** Regression coefficients of the ITS model for Araraquara x Araçatuba.

	coefficients Ω
(Intercept)	13.61** (4.92)
β_1_	0.18 *** (0.03)
β_2_	-14.32 (19.26)
β_3_	3.17* (1.30)
β_4_	-5.23 (6.95)
β_5_	-0.04 (0.04)
β_6_	96.37*** (27.24)
β_7_	-6.53 *** (1.84)
nobs	698
r.squared	0.21
adj.r.squared	0.20
sigma	44.22
statistic	26.58
p.value	0.00
df	8.00
*** p < 0.001; ** p < 0.01; * p < 0.05.	

Ω β_1_ describes the trend of the series for the control group before the institutional change, while β_2_ captures the change in level for the control group. β_3_ represents the change in the trend in the control group after lockdown. β_4_, the coefficient associated with the dummy variable G, indicates the difference between the treatment and control groups before the institutional change. In substantive terms, β_5_ represents the difference in the series trend before the intervention between the treatment and control groups. β_6_ can be interpreted as the difference in the series-level difference immediately after the intervention. The last coefficient of interest is β_7_, which indicates the difference in the trend of the series after the implementation of the social mobility restriction policy for the treatment group in relation to the control group.

## SUPPLEMENTARY MATERIAL

**TABLE 3:** Regression coefficients of the ITS model for Araraquara x São Carlos.

	coefficients Ω
(Intercept)	-5.61 (3.89)
β_1_	0.22*** (0.02)
β_2_	-8.61 (15.15)
β_3_	3.23** (1.03)
β_4_	13.99* (5.48)
β_5_	-0.07* (0.03)
β_6_	90.65*** (21.42)
β_7_	-6.60*** (1.45)
nobs	694
r.squared	0.34
adj.r.squared	0.33
sigma	34.76
statistic	49.88
p.value	0.00
df	8.00
*** p < 0.001; ** p < 0.01; * p < 0.05.	

Ω β_1_ describes the trend of the series for the control group before the institutional change, while β_2_ captures the change in the level of the control group. β_3_ represents the change in the trend in the control group after the lockdown. β_4_, the coefficient associated with the dummy variable G, indicates the difference between the treatment and control groups before the institutional change. In substantive terms, β_5_ represents the difference in the series trend before the intervention in the treatment and control groups. β_6_ can be interpreted as the difference in the series-level difference immediately after the intervention. The last coefficient of interest is β_7_, which indicates the difference in the trend of the series after the implementation of the social mobility restriction policy for the treatment group in relation to the control group.
